# Secular trend towards ultra-processed food consumption and expenditure compromises dietary quality among Taiwanese adolescents

**DOI:** 10.29219/fnr.v62.1565

**Published:** 2018-09-17

**Authors:** Yu-Chun Chen, Yi-Chen Huang, Yuan-Ting C. Lo, Hsing-Juan Wu, Mark L. Wahlqvist, Meei-Shyuan Lee

**Affiliations:** 1 School of Public Health, National Defense Medical Center, Taipei, Taiwan; 2 Department of Nutrition, China Medical University, Taichung, Taiwan; 3 Department of Food Nutrition, College of Human Science and Technology, Chung Hwa University of Medical Technology, Tainan, Taiwan; 4 Institute of Population Health Sciences, National Health Research Institutes, Zhunan Town, Miaoli, Taiwan; 5 Monash Asia Institute, Monash University, Melbourne, Victoria, Australia

**Keywords:** Ultra-processed food, adolescent, NOVA, NAHSIT, Taiwan, dietary quality

## Abstract

**Objective:**

To compare two Nutrition and Health Surveys in Taiwan (NAHSITs) 15–18 years apart to evaluate secular changes in ultra-processed food (UPF) consumption and expenditure among Taiwanese adolescents aged 16–18 years and the influences of such changes on dietary quality.

**Design:**

This cross-sectional study was based on two representative surveys (NAHSIT 1993–1996, *n* = 788; NAHSIT 2011, *n* = 1,274) of senior high school students. Dietary information and food expenditure were based on 24-h dietary recall. All food items were classified into original foods, processed culinary ingredients, processed foods, and UPFs based on NOVA criteria. Dietary quality was categorized as poor or good based on the mean of the Youth Healthy Eating Index–Taiwan Revised.

**Results:**

Compared to 1993–1996, adolescents consumed less energy from original foods (55 vs. 39%) but more from processed foods (12 vs. 18%) and UPFs (21 vs. 25%) in 2011, with no apparent gender differences. Those who consumed more UPFs had the lowest proportions of protein energy intake in both surveys (13.7 and 13.1%). Those who consumed more UPFs had higher levels of saturated fat and lower levels of monounsaturated and polyunsaturated fat, dietary fiber, and micronutrient intakes. The participants who consumed more UPFs and fewer original foods exhibited poorer dietary quality. Boys and girls exhibited equal UPF expenditure in both surveys despite an increase in UPF energy consumption. The adjusted odds ratios (95% confidence interval) were 1.33 (1.16–1.52) and 1.36 (1.10–1.69) for the risk of poor dietary quality with 10% increases in UPF energy intake and expenditure, respectively, in 2011.

**Conclusions:**

UPF energy consumption among Taiwanese adolescents increased between 1993–1996 and 2011. Observed trends in expenditure suggest that lower UPF costs influenced food choices during this period. Increasing UPF intake and expenditure was associated with poor dietary quality.

Food processing, which ranges from minimal to extensive processing, is defined as any procedure that alters food from its natural state (1). Food processing may affect patterns of purchase, use, and consumption as well as nutritional quality (including structure), affordability, and sustainability (2). Degree of processing has health implications arising from the lack of health protective food components like dietary fiber with decreased nutrient density; the greater presence of factors that are health adverse such as sodium, saturated and trans fatty acids, and contaminants including endocrine disruptors; and increased energy density (3, 4).

The implications of food processing are generally under-acknowledged. Growing evidence suggests that degrees of food processing are related to health outcomes (4–6). Highly processed products contain few or no recognizable whole foods and are referred to as *ultra-processed foods* (UPFs). The lack of recognizability reflects on the intactness of food and whether the basic commodity from which a food item or ingredient comes is difficult or impossible to identify (7). Excessive consumption of UPFs is believed to contribute to poor dietary quality and obesity (8, 9). UPFs are currently the subject of epidemiological investigations. The domestic acquisition of UPFs in various countries has increased in recent decades (10–12). Information about UPF consumption, its costs, and its health effects is not readily available in Asia.

Groups particularly vulnerable to this changing food system exposure include adolescents as they form lifelong eating patterns relevant to their own health and that of the generations that will follow them (8, 13–15). Nutrition and Health Surveys in Taiwan (NAHSITs) provide the opportunity to evaluate the hypothesis that changes in UPF consumption and expenditure among Taiwanese adolescents affect dietary quality. We compared two senior high school student surveys conducted 15–18 years apart to evaluate this hypothesis.

## Methods

### Study population

The NAHSITs are an established policy instrument of the Center for Survey Research, Academia Sinica, on behalf of the Department of Health, Taiwan, since 1993. They survey representative samples of the free-living population. The present study investigated two representative NAHSITs conducted, among others, on senior high school students aged 16–18 in 1993–1996 (household-based students only, not including those who were not at school) and 2011 (school-based). The design and sampling of both surveys have been described in previous studies (16, 17). Both surveys adopted a multistaged, stratified, and clustered probability design. In the 1993–1996 household-based survey, there were five mutually exclusive strata (Northern 1, Northern 2, Central, Southern, and Eastern strata) and three extra strata for specific population groups (Hakka areas, Penghu Islands, and mountainous areas). For the age group 16–18, 8–12 people were randomly selected from each stratum. The 2011 survey comprised five mutually exclusive strata (Northern 1, Northern 2, Central, Southern, and Eastern strata), and from each of these strata four senior high schools were randomly selected; in turn, 60 students were randomly selected from each high school. The 1993–1996 survey was completed by 391 boys and 397 girls. In 2011, 633 boys and 641 girls participated. All participants had face-to-face interviews to provide sociodemographics and health examinations for anthropometry and fasting blood collection.

### Measurements

#### Dietary intake and dietary quality index

Dietary information was obtained through interviews. The proportions of daily energy intake from each category as well as nutrient densities (per 1,000 kcal) for selected macro- and micronutrients were calculated based on each participant’s 24-h dietary recall (18) by using local food composition tables (19). Enquiry was made of participants about foods consumed in the past 24 h, with timing, location, and food source. To aid the recall process, food models, measuring cups, spoons, and cue cards were used. Food intake data were expressed as weights of foods consumed, taking into account individual recipes and condiments (including cooking oil, salt, and sauces) used (20).

The overall dietary quality of the participants was assessed by the Youth Healthy Eating Index–Taiwan Revised (YHEI-TwR-90), which uses information from the 24-h dietary recall and a food frequency questionnaire to derive a score modified from the US YHEI (21, 22). The YHEI-TwR-90 scores were based on daily consumption of the following 10 items: 1) whole grains (0 to ≥2 servings, 0–10 points), 2) vegetables (0 to ≥3 servings, 0–10 points), 3) fruits (0 to ≥3 servings, 0–10 points), 4) dairy (0 to ≥3 servings, 0–10 points), 5) meat ratio (0 to ≥20, 15–0 points), 6) snack foods (i.e. salty snacks and snacks with added sugar, 0 to ≥3 servings, 10–0 points), 7) sweetened beverages (0 to ≥3 servings, 10–0 points), 8) fried foods outside the home (never to daily, 5–0 points), 9) breakfast (never to >5 times/week, 0–5 points) and 10) dinner (never to >5 times/week, 0–5 points). Scores were proportionate to consumption and in aggregate ranged from 0 to 90, with higher scores indicating better dietary quality. For sensitivity analysis, a YHEI-TwR-70 was formed by removing snack foods and sweetened beverages. We defined good and poor dietary quality depending on whether a participant’s score was lower or higher than the mean.

#### Food classification

Moubarac et al. (7) define UPFs as foods ‘formulated mostly or entirely from substances derived from foods. [But] typically [they] contain little or no whole foods … Numerically the majority of ingredients [in UPFs] are preservatives … processing aids and other additives … [UPFs] displace food-based freshly prepared dishes, meals. Processes [in their production] include hydrogenation, hydrolysis; extruding, molding, reshaping; pre-processing by frying, baking’. An earlier paper by the same investigators makes the case for a classification of foods in accordance with the extent and purpose of processing (23).

**Fig. 1:**
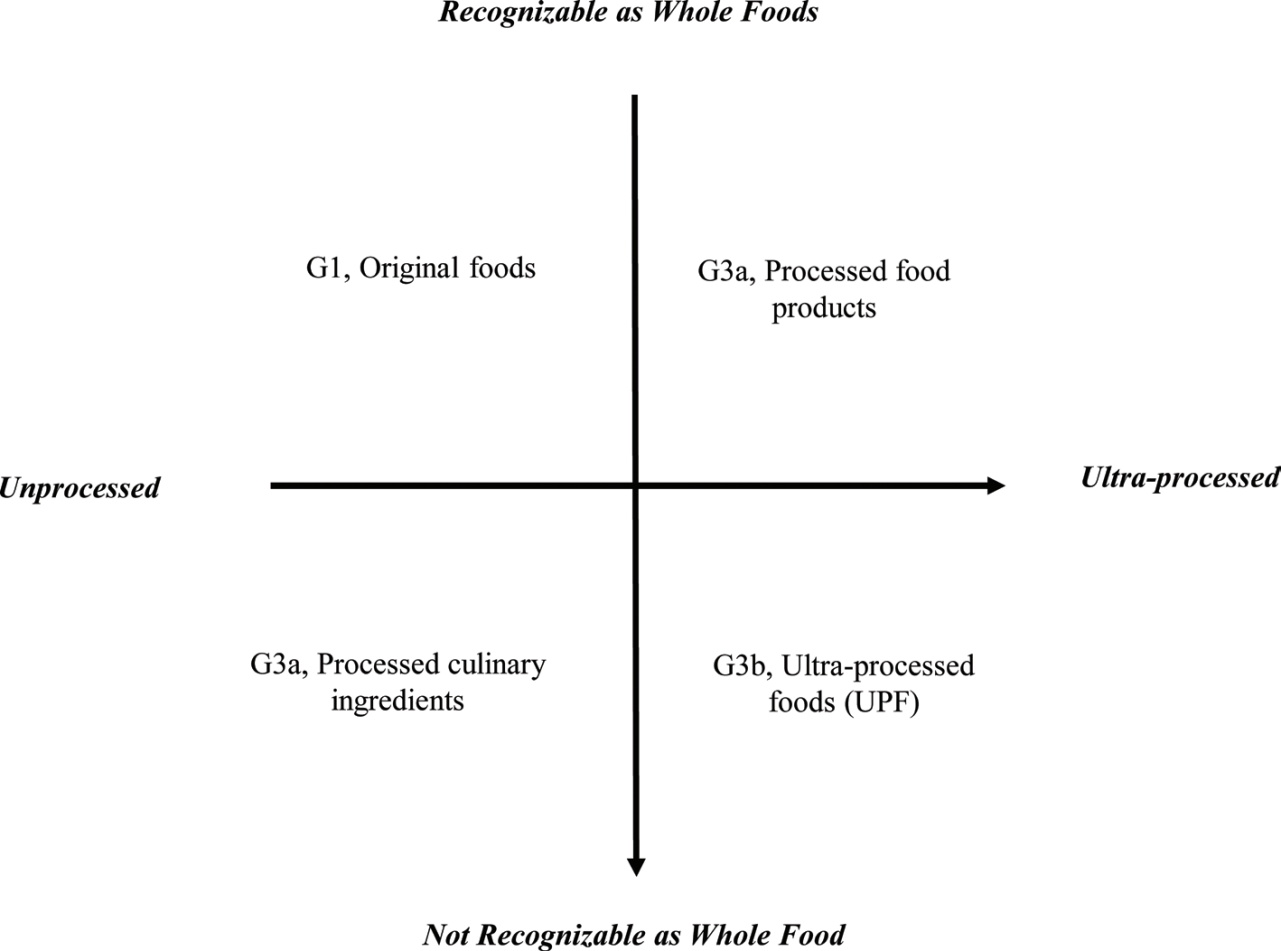
NOVA classification system.

On this basis, in the present study, foods were classified according to two dimensions (Fig. 1). The first dimension was the degree of processing, ranging from ‘unprocessed’ to ‘ultra-processed’. The second was whether the **structures of the original foods were recognizable**. All food items reported in the two surveys were categorized into three principle groups and subgroups according to the degree of processing based on the NOVA [a name, not an acronym (24)] criteria (17). Group 1 (G1) comprised original foods, e.g. rice, fresh fish, fruits and vegetables, dried beans, fresh milk; and Group 2 (G2) comprised processed culinary ingredients, e.g. olive oil, sugar and salt, starch and flour. Group 3 (G3) comprised ready-to-eat products and was divided into two subgroups – processed foods (G3a), e.g. tinned or canned fish, ham, bread, pickles, and UPFs (G3b), e.g. hot dogs, chips, instant noodles, sweetened milk drinks. This food classification system did not include alcoholic beverages and condiments, which were classified as ‘others’.

Traditional Taiwanese snacks such as dumplings, stinky tofu, oyster omelets, pan-fried buns, blood *pudding*, and processed meat and fish that were homemade or sold by local street vendors were included in G3a.

See supplementary table for examples.

#### Food expenditure calculation

The food expenditure calculation, based on the 24-h recall of individual participants in this study, has been published previously (25). In summary, food retail prices were identified from governmental websites to analyze retail prices during the survey periods. National average monthly prices at survey time were used to calculate food costs on the interview dates. Prices were supplemented and adjusted based on supermarket chain price fluctuations, which were discounted and deflated based on the consumer price index at the times of the two surveys. For each food, expenditure was calculated by multiplying the weight of the purchased item by the price of that particular food. No adjustments were made for eating at restaurants or celebrations. Participants’ food expenditure was generated from these food costs with the assumption that the food was eaten at home.

### Statistical analysis

Means and standard errors were reported for continuous variables such as anthropometric measures, total and relative energy intake, and food expenditure as percentages among the food groups. The percentages are reported for categorical variables. A one-way analysis of variance (ANOVA) was used to compare the relative contributions of the food groups to energy and expenditure in the two surveys, and participants were further classified based on UPF energy intake quartiles. The ANOVA and linear regression analyses were used to assess linear trends in macro- and micronutrient densities against UPF consumption. Logistic regression was used to evaluate the effects of UPF energy intake and expenditure on dietary quality (good or poor) for the 2011 survey. We fixed the total energy intake and energy intake percentages in G1 and G3a while examining UPFs and dietary quality. The control variables for UPF expenditure and dietary quality were total food expenditure and G1 and G3a expenditures. In Model 2, both analyses further controlled for gender, grade, locality, major caregivers, household income, household expenditure, mother’s education, smoking, alcohol drinking, and body mass index (BMI). Data were weighted to represent the adolescent population in Taiwan in the two surveys. All analyses were performed using the SAS software version 9.1.3 (SAS Institute Inc., Cary, NC, USA). SUDAAN version 10.0 (RTI International, Research Triangle Park, NC, USA) was used to adjust for the design effect of sampling.

## Results

Table 1 shows the participant characteristics for the two surveys. A minor shift in the ethnicity profile was observed between the two, with fewer Fukienese individuals and more of each of the other groups observed in the 2011 survey. Overall, dietary quality as judged by YHEI-TwR (both 90 and 70 sets) did not change significantly. Body-fat indices increased between the two surveys. In particular, BMI increased by 0.8–1.6 units along with increased abdominal and truncal fatness. Based on the criteria in Taiwan, obesity prevalence more than doubled from 7.5 to 16.8% and lipoproteins presented a greater cardiovascular risk in the second survey. When body fatness was considered by way of WHO BMI criteria, the trend was similar.

**Table 1:** Participant characteristics of the 1993–1996 and 2011 NAHSITs^a^

	NAHSIT 1993–1996			NAHSIT 2011		
	Total	Boys	Girls	Total	Boys	Girls
*n*	788	391	397	1,274	633	641
YHEI-TwR-90^b^	46.1 ± 0.6	46.2 ± 1.0	46.1 ± 1.0	45.3 ± 0.8	44.7 ± 0.8	46.0 ± 0.8
YHEI-TwR-70^c^	38.3 ± 0.5	38.1 ± 0.9	38.5 ± 1.0	37.2 ± 0.8	37.1 ± 0.8	37.2 ± 0.9
Age (years)	16.7 ± 0.1	16.6 ± 0.1	16.7 ± 0.1	16.6 ± 0.04	16.6 ± 0.1	16.5 ± 0.04
Ethnicity (%)						
Fukienese	79.8	80.1	79.4	73.6	70.2	77.5
Hakka	9.9	9.6	10.3	12.1	13.3	10.8
Mainlander	9.0	8.8	9.2	10.7	12.5	8.7
Indigenous	1.3	1.5	1.1	3.5	4.0	3.0
BMI (kg/m^2^)	20.8 ± 0.2	21.0 ± 0.3	20.6 ± 0.2	22.0 ± 0.1	22.6 ± 0.2	21.4 ± 0.2
Underweight						
WHO std^d^	20.1	22.5	30.9	27.4	16.5	21.7
Taiwan std^e^	13.3	8.5	18.3	8.5	8.1	8.8
Normal						
WHO std	62.7	65.0	59.9	61.3	59.2	63.6
Taiwan std	66.6	73.1	60.1	61.4	59.3	63.8
Overweight						
WHO std	12.3	10.1	6.3	7.9	15.7	10.7
Taiwan std	12.5	10.5	14.6	13.4	12.9	13.9
Obese						
WHO std	4.9	2.4	2.9	3.3	8.6	4.0
Taiwan std	7.5	7.9	7.1	16.8	19.7	13.5
TSF (cm)	15.0 ± 0.6	11.3 ± 0.4	18.8 ± 0.6	17.7 ± 0.3	14.6 ± 0.4	21.0 ± 0.4
WC (cm)	66.9 ± 0.4	69.7 ± 0.6	64.1 ± 0.5	78.1 ± 0.3	79.6 ± 0.5	76.5 ± 0.4
TG (mg/dL)	67.0 ± 2.4	66.9 ± 2.7	67.0 ± 3.3	72.4 ± 1.1	74.0 ± 2.0	70.6 ± 1.2
LDL-C (mg/dL)	91.6 ± 2.1	88.9 ± 2.7	94.2 ± 2.4	91.7 ± 0.9	89.9 ± 1.1	93.7 ± 1.3
HDL-C (mg/dL)	57.7 ± 2.0	54.9 ± 1.8	60.2 ± 2.8	55.1 ± 0.4	51.6 ± 0.6	59.0 ± 0.4

NAHSIT, Nutrition and Health Survey in Taiwan; TSF, triceps skinfold thickness; WC, waist circumference; TG, triglycerides; LDL-C, low-density lipoprotein cholesterol; HDL-C, high-density lipoprotein cholesterol; YHEI-TwR, Youth Healthy Eating Index–Taiwan Revised.

a Values are weighted to reflect their representation in the population and are percentages of mean ± SE.

b YHEI-TwR-90 total scores range from 0 to 90, with higher scores indicating higher dietary quality.

c YHEI-TwR-70 scores not including soft drinks and snacks range from 0 to 70.

d WHO standards (http://www.who.int/growthref/who2007_bmi_for_age/en/)

e Taiwanese standards (https://obesity.hpa.gov.tw/TC/BMIproposal.aspx)

The changes in the proportions of each of the three main food groups constituting the average daily energy intake from 1993–1996 to 2011 are shown in Table 2. Adolescents gained less daily energy from original foods (G1; 55 to 39%) but more from processed foods (G3a; 13 to 18%) and UPFs (G3b; 21 to 25%) in 2011. In addition, the contribution of alcoholic beverages and condiments increased from 3 to 5% of energy intake (data not shown).

**Table 2:** Percentages of daily dietary energy intake and food expenditure for four food groups in the two surveys^a^

	Total			Boys			Girls		
	Year of survey			Year of survey			Year of survey		
	1993–1996	2011	*P*^b^	1993–1996	2011	*P*^b^	1993–1996	2011	*P*^b^
*n*	788	1,274		391	633		397	641
Total energy intake (kcal/day)		
2,059 ± 73.8	2,416 ± 59.1	0.003	2,555 ± 127	2,752 ± 77	0.222	1,563 ± 79.1	2,043 ± 49	<0.001
Percent of energy intake^c^		
G1	55.3 ± 0.8	38.8 ± 0.8	<0.001	57.5 ± 1.7	41.5 ± 1.0	<0.001	53.0 ± 1.2	35.8 ± 1.1	<0.001
G2	7.63 ± 0.8	13.0 ± 0.5	<0.001	7.10 ± 0.4	13.1 ± 0.6	<0.001	8.15 ± 1.4	12.9 ± 0.7	0.009
G3a	13.0 ± 0.4	17.8 ± 0.7	<0.001	13.0 ± 1.2	17.0 ± 0.9	0.022	13.0 ± 1.2	18.8 ± 1.0	0.004
G3b	21.5 ± 1.1	25.5 ± 0.6	0.011	19.5 ± 1.9	23.5 ± 0.8	0.083	23.6 ± 1.2	27.6 ± 0.8	0.018
Percent of expenditure on food^c^		
G1	64.2 ± 1.3	58.6 ± 1.0	0.005	64.1 ± 2.0	60.4 ± 1.1	0.133	64.2 ± 1.6	56.6 ± 1.4	0.005
G2	1.54 ± 0.3	4.75 ± 0.2	<0.001	1.79 ± 0.5	4.48 ± 0.3	0.001	1.29 ± 0.2	5.07 ± 0.3	<0.001
G3a	11.1 ± 1.3	13.4 ± 0.5	0.127	11.6 ± 1.3	11.6 ± 1.3	0.326	10.5 ± 1.5	13.8 ± 0.8	0.081
G3b	23.7 ± 1.0	23.2 ± 0.7	0.697	23.0 ± 2.0	22.1 ± 0.9	0.698	24.4 ± 1.0	24.5 ± 0.8	0.964

G1, original foods; G2, processed culinary ingredients; G3a, ready-to-eat processed foods; G3b, ready-to-eat ultra-processed foods.

a Values are weighted to reflect their representation in the population and are calculated by mean ± SE.

b Calculated by one-way analysis of variance.

c Data are the mean ± SE of percentages of daily energy intake and food expenditure.

**Table 3:** Distributions of daily macronutrient intakes and nutrient densities based on UPF consumption from NAHSIT 2011^a^

	Total	Quartiles of UPF consumption^b^				*P*^c^	β^d^
		Q1	Q2	Q3	Q4		
UPF energy (kcal/d)	615	135	436	724	1,163		
Percent energy (%)
Carbohydrates	53.0	50.6	51.7	52.8	56.9	<0.001	0.20
Protein	15.1	16.7	15.7	14.8	13.1	<0.001	−0.12
Fat	32.4	33.0	32.9	32.8	30.7	0.004	−0.07
Nutrient density (per 1,000 kcal/day)
SFA (g)	12.4	11.5	12.2	12.9	13.0	<0.001	0.05
MUFA (g)	12.6	13.6	12.8	12.5	11.4	<0.001	−0.07
PUFA (g)	10.9	11.6	11.5	11.0	9.50	<0.001	−0.07
Vitamins
Vitamin A (RE)	289	368	331	231	225	0.004	−5.30
Vitamin E (mg)^e^	3.75	3.92	3.81	3.81	3.45	0.193	−0.01
Vitamin C (mg)	51.6	58.1	52.1	47.1	49.1	0.040	−0.32
Vitamin D (μg)	2.06	2.48	2.05	1.90	1.81	0.017	−0.02
Vitamin B-1 (mg)	0.60	0.68	0.66	0.57	0.51	<0.001	−0.01
Vitamin B-2 (mg)	0.60	0.6	0.62	0.58	0.58	0.155	−0.001
Niacin (mg)^f^	8.93	9.65	9.49	8.82	7.77	<0.001	−0.06
Vitamin B-6 (mg)	0.75	0.85	0.78	0.75	0.63	<0.001	−0.01
Vitamin B-12 (μg)	2.13	2.45	2.09	1.92	2.07	0.615	−0.01
Minerals
Potassium (mg)	987	1,089	1,007	949	905	<0.001	−6.12
Magnesium (mg)	108	120	109	104	98.0	<0.001	−0.71
Calcium (mg)	215	226	219	210	203	0.100	−0.80
Iron (mg)	7.36	7.88	7.61	7.14	6.83	0.037	−0.04

UPF, ultra-processed food; SFA, saturated fatty acids; MUFA, mono-unsaturated fatty acids; PUFA, poly-unsaturated fatty acids; vitamin B-1, thiamin; vitamin B-2, riboflavin.

a Values are weighted to reflect their representation in the population.

b UPF does not include alcoholic beverages or condiments.

c A one-way ANOVA was conducted to test for differences in the UPF consumption energy ratio group.

d Regression coefficient of the dietary micronutrient content in 10% of the total dietary caloric value from UPFs.

e α-TE.

f Preformed only.

The trends for gender-specific percentages of daily dietary energy for the food groups classified based on degrees of processing were similar to those of the whole population. Of interest, daily energy intake in girls, but not boys, increased significantly from 1,563 kcal in 1993–1996 to 2,043 kcal in 2011. Boys’ UPF consumption increased from 498 kcal to 646 kcal (by 29.7%) and girls’ from 368 kcal to 564 kcal (by 53.3%). These increases correspond with increases in BMI (Table 1). Although both boys and girls gained more energy from UPFs in 2011, UPF expenditure did not change. Girls reduced their original food (G1) expenditure but increased that of processed culinary ingredients (G2) and processed foods (G3a).

Table 3 shows that the daily total energy intake from protein was progressively less with the higher UPF consumption energy quartiles in 2011. Those in the highest UPF quartile exhibited the lowest protein energy intake. Similar relationships were observed for degrees of intake for monounsaturated fat, polyunsaturated fat, and dietary fiber. By contrast, every 1,000 kcal of energy from saturated fat ranged from 11.5 g in the lowest quartile to 13.0 g in the highest. Negative associations were observed between the relative consumption of UPFs and micronutrient intake based on nutrient densities (vitamins A, C, D, B-1, and B-6, as well as niacin, potassium, magnesium, calcium, and iron). Similar findings were evident in 1993–1996 (data not shown).

Table 4 shows the associations between UPF energy intake and expenditure and dietary quality in 2011. A 10% increase in UPF energy intake or expenditure was associated with an increased risk of poor dietary quality by 37 or 36%. Regarding further control for potential covariates, the odds ratios (95% confidence interval) of poor dietary quality were 1.33 (1.16–1.52) for a 10% increase in UPF energy and 1.36 (1.10–1.69) for a 10% increase in UPF expenditure.

**Table 4:** Odds ratios (95% CI)^a^ for risk of poor dietary quality with a 10% increase in UPF energy intake or expenditure from NAHSIT 2011 (*N* = 817,658; *n* = 1,274)

YHEI-TwR-90 Poor vs. good^b^	10% increase	
	UPF energy intake	UPF expenditure
Crude	1.37 (1.22–1.54)	1.36 (1.26–1.47)
Model 1	1.21 (1.05–1.39)^c^	1.35 (1.15–1.59)^d^
Model 2	1.33 (1.16–1.52)^e^	1.36 (1.10–1.69)^e^

YHEI-TwR-90, Youth Healthy Eating Index–Taiwan Revised

a Logistic regression was used to assess odd ratios (95% CI).

b YHEI-TwR-90 total scores range from 0 to 90, with higher scores indicating higher dietary quality. The threshold between poor and good dietary quality was based on the mean score.

c Model 1 was adjusted for the percentages of energy intake in G1 and G3a and total energy intake.

d Model 1 was adjusted for food expenditure in G1 and G3a and total food expenditure.

e Model 2 was further adjusted for gender, grade, locality, major caregivers, household income, household expenditure, mother’s education, smoking, alcohol drinking, and BMI.

## Discussion

We found that the consumption of UPFs among Taiwanese senior high school students increased, leading to a reduction in dietary quality between 1993–1996 and 2011. The observed trends in expenditure on food suggest that lower UPF costs influenced food choices during this period. Increasing UPF intake and expenditure was associated with poor dietary quality.

### Changing dietary patterns

The current study provides a unique description of changes in the consumption of industrially processed foods among Taiwanese adolescents from 1993–1996 to 2011. Original foods are now less of a staple for adolescents. Dietary changes are characterized by the reduced consumption of original foods used in the preparation of dishes and meals; such foods have been replaced by ready-to-eat processed foods and UPFs, thereby contributing to concerns already evident in various parts of the world where the consumption of such foods has also increased (10–12, 26–28). An increased presence of UPFs can be observed in the global food system and people’s diets (29–31). According to data from the National Health and Nutrition Examination Survey in the United States, in 2007–2008, 41% of adolescents consumed UPFs from fast-food restaurants (32). This phenomenon is of potential concern to people of all ages. In Canada, the contribution of ready-to-eat products to people’s dietary energy increased from 28.7 to 61.7% from 1938 to 2011, thereby underscoring the importance of considering food processing in dietary assessments (12). In addition, the consumption of UPFs in Sweden increased by 142% between 1960 and 2010, and the associated prevalence of obesity increased from 5 to 11% (10). Similarly, in a nationally representative sample in the United States, energy intake contributions from moderately and highly processed food products provided more than a quarter of all dietary energy among the population in 2012 (1). Similarly, in 2011, Taiwanese boys and girls consumed 23.5 and 27.6%, respectively, of food energy from UPFs.

UPFs are part of a global food system (31). Several factors are likely to be driving the increased consumption of UPFs worldwide, including rising household incomes, rapid urbanization, and aggressive commercialization (29). UPFs may not be harmful when consumed in small amounts but excessive consumption could compromise the ability to be sated, encourage energy overload, and lead to obesity and related health problems (31). In Brazil, high levels of UPF consumption are associated with the metabolic syndrome in adolescents (6). UPFs are often devoid of cultural specificity, designed for universal markets as hyperpalatable items, and presented specifically to attract adolescents, thereby popularizing unhealthy food choices (33). The association between UPF intake and overweight and obesity among Brazilian adolescents and adults is evident (9). We found that the increase in UPFs was accompanied by increased BMIs as well as obesity rates in both Taiwanese boys (7.9 to 19.7%) and girls (7.1 to 13.5%) during the 18-yr period from 1993–1996 to 2011.

The trend towards higher UPF intake in Taiwan observed in the present study may be a forerunner of adverse health outcomes. However, no statistically significant association between UPF intake and overweight was observed in this study (data not shown), possibly because a critical intake threshold has not been reached or because other factors counteract the risk. Nevertheless, dietary transition is underway in Taiwan in terms of not only UPFs but also the increased consumption of processed culinary ingredients such as edible oils, sugars, starches, and flour. Such trends erode traditional food culture, such as the use of refined wheat flour in rice-based cultures, which enables the advent of bakery items, snacks, burger buns, noodles, cakes, and biscuits. Such changes are reflected in the changing dietary habits of young Taiwanese observed in a previous study that compared the NAHSIT results between 1993–1996 and 2005–2008 (34).

### Dietary quality and UPFs

We found that eating more UPFs and fewer original foods, leading to greater energy density and less nutritious diets, resulted in more adverse health risk among adolescents, especially regarding cardiometabolic risk as judged by waist circumference and lipoprotein status (4, 6, 10, 26, 35, 36). Adolescence is one of the most dynamic and complex periods of transition in a human’s life span (13). Adolescents are vulnerable to risk-taking behaviors such as those related to food choices because of identity seeking, career-building, uncertain relationships, and financial constraints. However, adolescence is a period of nutritional scene-setting for reproductive and working adult lives involving family responsibilities. For these and various other reasons, excessive intake of UPFs may be of great significance for the health and well-being of current and future generations (14, 15).

To achieve the World Health Organization goal of reducing premature mortality due to noncommunicable diseases by 25% by 2025, the production of UPFs must be actively addressed (37); otherwise people of all ages will be increasingly more exposed to unhealthy foods that are energy dense, of poor nutritional quality, high in salt, and vehicles for trans-fatty acids (38). Unfortunately, rather than using basic food commodities, reformulation often allows manufacturers to advertise their products as ‘healthy’ in limited respects (e.g. high in fiber, low in salt and fat) when they are not legitimate ‘healthy alternatives’. In addition, we found that the increased consumption of such products is likely to at least maintain, if not displace, the consumption of original foods. This would lead to a reduction in food biodiversity, which is the most universally accepted and crucial of all the food-based dietary guidelines (38–40). Food diversity is positively related to health and survival (41) and ultra-processing destroys the structure of food, which is a critical determinant of health outcomes. Although dietary fiber may be isolated from food and subsequently added back as an ingredient, it does not fulfil all of its functional potential after its original structural characteristics have been replaced by others perceived to be less healthy (2). Hence, original foods are generally healthier than processed foods. Nevertheless, there may be some benefits in using the accepted nutrient or health claims in, for example, European or national legislation, within specific food categories.

A generally accepted index of dietary quality in children (YHEI-TwR) did not detect a shift in dietary pattern between the two surveys studied (Table 1). However, the YHEI-TwR was associated with increases in energy intake and expenditure from UPF. Moreover, UPF intake evidently increased during the period in question, indicating that direct assessments of less nutritious foods are required for the evaluation of nutritionally related health in children. Furthermore, recognizing when indices of cardiometabolic health such as body fatness have deteriorated over time is crucial.

### Food price, expenditure, and choice

We found that UPF expenditure did not increase over the 15–18 year time frame of this study; however, UPF energy consumption did. This indicates that UPFs became relatively cheaper during this period, which provides some insight into factors that might be encouraging their consumption, namely cost and palatability. In addition, this reduction in relative cost might be consistent with added profit margins for suppliers. However, girls spent less on original foods and more on processed culinary ingredients such as sugar and oil than did boys (Table 2). Such complex choices may be a result of limited budgets or uncertain health-seeking behaviors.

### Strengths and limitations

The present study has several strengths. First, the results are representative of senior high school students throughout Taiwan, thereby enabling extrapolation to this demographic group with reasonable confidence. Given cultural similarities, the results could represent other Northeast Asian countries to a certain extent. Indeed, trends in this region seem to be similar to those in other advanced or transitional economies. In addition, similarly to countries of dominantly European ancestry, increased UPF consumption may develop into cardiometabolic risk.

The first limitation of the present study is that the method it used, 24-h dietary recall, to estimate daily intake may have underestimated or overestimated actual consumption. A larger sample size and higher representativeness would limit this problem. The classification of UPF by NOVA has come to be a reference method (23). However, its cross-cultural extrapolation requires assumptions that may not be valid or feasible, with consequent misclassification of food groups. Another limitation was the study design; despite comparable sampling, the first and second surveys may have been asymmetrical. Moreover, no conclusions regarding cause and effect can be drawn. Nevertheless, the purpose of this study was to examine and draw conclusions regarding secular trends, which the design allowed for.

## Conclusions

We provide evidence that ready-to-eat food products have yielded changes in Northeast Asian adolescent dietary patterns from 1993–1996 to 2011, especially in terms of UPFs partially replacing original foods and traditional dishes and meals. The adolescents investigated in this study who consumed more UPFs and fewer original foods had less healthy nutrient and cardiometabolic risk profiles without having incurred additional expenditure on foods. These findings indicate that national health system planning, and policy-making, should be more responsive to significant changes occurring in the food system, related nutritional and health impact trajectories, and cost factors in food choices.

## Supplementary Material

Secular trend towards ultra-processed food consumption and expenditure compromises dietary quality among Taiwanese adolescents
