# Supplementary material for: Secular trend towards ultra-processed food consumption and expenditure compromises dietary quality among Taiwanese adolescents
Source: Food Nutr Res. 2018 Sep 17;62:10.29219/fnr.v62.1565. doi: 10.29219/fnr.v62.1565 (PMC6150927; doi:10.29219/fnr.v62.1565)
Supplement: Secular trend towards ultra-processed food consumption and expenditure compromises dietary quality among Taiwanese adolescents [file FNR-62-1565-s001.pdf]

**Supplementary Table.** Taiwanese food classification based on the extent of processing

| Food group | Extent and purpose of processing | Examples |
|---|---|---|
| Group 1 (G1) | Unprocessed or minimally processed foods | Rice, grains and dried beans; fresh fruit and vegetables, fruit or vegetable juices without added sugar, fish and seafood, meat, poultry; eggs; milk (pasteurized, fermented or powdered), etc.<br>**Specific Taiwanese foods** (homemade or street food): savory sticky rice, rice dumpling wrapped with bamboo leaves, rice ball, sushi, popped rice, popiah rolls, oyster vermicelli, Aiyu jelly, etc. |
| Group 2 (G2) | Processed culinary ingredients | Vegetable oils (e.g., crushed from soybeans, peanuts or sesame seeds), animal fats (e.g., lard or butter); starches extracted from sweet potatoes or other plants; wheat flour and noodles, rice noodles, mung bean noodles, tapioca bubbles; sugar, honey, etc. |
| Group 3a (G3a) | Processed foods | Canned or bottled vegetables, fruits or beans; canned fish; salted, cured, or smoked meats; sausage; dried fruit or vegetables, etc.<br>**Specific Taiwanese foods**: soybean products (e.g., soy drink, tofu, dried tofu, stinky tofu); radish cake, dumpling, pork bun, pan-fried buns, oyster omelets, blood pudding, pork jerky, pork floss, steamed bread, Chinese style baked roll, fried bread stick, etc. |
| Group 3b (G3b) | Ultra-processed food and drink products; ready to eat, to drink or to heat, including microwaveable foods | Pre-prepared or packaged pies, red bean bun, pizza, fish ball, fish cake, dumplings ; sugary beverages; sweetened milk drinks; energy bars; fast foods (e.g., burgers, French fries, hot dogs, chicken nuggets); breakfast cereals, corn or potato chips, ice cream, chocolate, candies, etc. |
